# Nano-Sized Fucoidan Interpolyelectrolyte Complexes: Recent Advances in Design and Prospects for Biomedical Applications

**DOI:** 10.3390/ijms24032615

**Published:** 2023-01-30

**Authors:** Natallia V. Dubashynskaya, Ekaterina R. Gasilova, Yury A. Skorik

**Affiliations:** Institute of Macromolecular Compounds, Russian Academy of Sciences, Bolshoi VO 31, St. Petersburg 199004, Russia

**Keywords:** fucoidan, chitosan, polysaccharides, interpolyelectrolyte complexes, drug delivery systems

## Abstract

The marine polysaccharide fucoidan (FUC) is a promising polymer for pharmaceutical research and development of novel drug delivery systems with modified release and targeted delivery. The presence of a sulfate group in the polysaccharide makes FUC an excellent candidate for the formation of interpolyelectrolyte complexes (PECs) with various polycations. However, due to the structural diversity of FUC, the design of FUC-based nanoformulations is challenging. This review describes the main strategies for the use of FUC-based PECs to develop drug delivery systems with improved biopharmaceutical properties, including nanocarriers in the form of FUC–chitosan PECs for pH-sensitive oral delivery, targeted delivery systems, and polymeric nanoparticles for improved hydrophobic drug delivery (e.g., FUC-zein PECs, core-shell structures obtained by the layer-by-layer self-assembly method, and self-assembled hydrophobically modified FUC particles). The importance of a complex study of the FUC structure, and the formation process of PECs based on it for obtaining reproducible polymeric nanoformulations with the desired properties, is also discussed.

## 1. Introduction

Polysaccharides, such as alginate, fucoidan (FUC), carrageenan, etc., are structural components of the cell walls of marine algae; they have been widely used in drug development due to their unique physicochemical and biological properties [1,2,3,4,5]. Drug modification by developing various polymeric drug delivery systems is a promising strategy in modern pharmaceutical research and development (R&D) to improve the biopharmaceutical properties of drugs (increased bioavailability and modified (sustained, controlled, and targeted) drug release) and reduce their side effects [6]. One way to create such polymeric structures is to obtain different interpolyelectrolyte complexes (PECs) [7].

Recently, researchers have focused their attention on a polysaccharide FUC, which has a number of useful properties (e.g., emulsifier and viscosity enhancer) [8], as well as a broad spectrum of biological activities (antitumor [9,10,11,12,13], antiviral [14,15], antioxidant [16], anti-allergic [17], anti-inflammatory [18], anti-diabetic and hypoglycemic [19], immunoregulatory [20], cardio- and cardiovascular protective [21,22], renoprotective [23], and hepatoprotective effects [24]). In addition, FUC can interact with macrophages, growth factors, cytokines, and P-selectin, as well as inhibit the P-glycoprotein pump and α-glucosidase, and increase intestinal permeability by improving paracellular transport [2,25,26,27]. In addition, FUC is a non-toxic, biodegradable and biocompatible polymer [28,29,30] that has been approved by the U.S. Food and Drug Administration as a food ingredient, under the category “Generally Recognized as Safe” [31]. This makes FUC an attractive excipient for pharmaceutical R&D.

FUC is a sulfated marine polysaccharide usually extracted from brown algae [1]; the pharmacological effects of FUC depend on its physicochemical properties, mainly on its molecular structure, monosaccharide composition, degree and position of sulfation, branching, and molecular weight (MW) [13,19,32]. Due to its anionic nature, it is possible to assemble FUC into interpolymeric structures (e.g., nanoparticles (NPs) in the form of polyelectrolyte complexes, as well as nanogels, cross-linked gels, etc.) with both positively charged polymers (synthetic and natural) and with low-MW biologically active compounds [33,34]. The structural diversity of FUC influences both the physicochemical (e.g., hydrodynamic size, surface charge, and colloidal stability) and the biopharmaceutical (including loading efficiency and encapsulation efficiency, and drug release profile) properties of interpolymeric formulations [35,36]. Therefore, it is important to consider the influence of the properties of the initial polymers in the developing drug delivery systems, in the form of PECs with reproducible parameters.

A limited number of reviews have been published on the various biomedical applications of FUC and its nanostructures. For example, Ramos-de-la-Peña et al. [33] described the extraction and purification of FUC and its structural and bioactive role in the preparation of nanogels for loading various bioactive compounds. Venkatesan et al. [1] reviewed various methods for obtaining different types of polymeric FUC NPs (e.g., using microwave, emulsion, solvent evaporation, green synthesis, polyelectrolyte self-assembly, precipitation, and ultrasonication methods) including FUC composites with metal NPs. Iqbal et al. [37] focused on obtaining FUC-based composites in the form of bioactive films and coatings for medical and food applications. Tran et al. [38] described various drug delivery nanostructures (e.g., pH-sensitive NP, biphasic-release NP, multilayer nanocapsules, and nanocomposite scaffolds) based on FUC, including its combination with chitosan (CS), as well as the use of FUC for surface functionalization of metal NPs. Barbosa et al. [39] provided an overview of FUC and CS, including their sources, chemical structure, biological properties, and medical use, as well as their combination, in the form of NPs, to deliver different classes of compounds and materials for tissue engineering.

This review discusses the use of polyanionic FUC to prepare PECs with polycations of different natures for the development of safe and effective drug delivery systems. We focused on key aspects of the preparation and investigation of different types of PECs (e.g., polyelectrolyte particles, core-shell NPs, multilayer NPs, and self-assembling polymer structures) with stable and reproducible properties, such as the comprehensive characterization of the FUCs used, as well as details of the technological procedure (including concentrations and ratios of polymers, pH of polymer solutions, and mixing order of ingredients).

## 2. Chemical Structure of FUC

**Figure 1:**
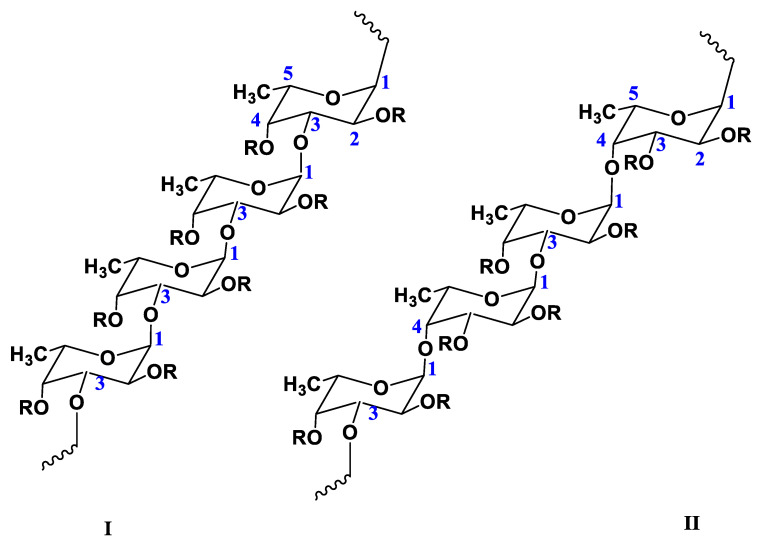
Two types of homofucose backbone chains in brown seaweed fucoidans. Chain (**I**) contains (1→3)-α-L-fucopyranose, chain (**II**) contains alternating (1→3)- and (1→4)-α-L-fucopyranose residues; R—carbohydrate substituents (sugars) or noncarbohydrate substituents (sulfate or acetate groups).

FUC is a sulfated polysaccharide structurally based on the monosaccharide α-L-fucose [40]. The main sources of FUC are marine brown algae (*Phaeophyta*, *Laminariaceae*, *Fucaceae*, *Chordariaceae*, and *Alariaceae*) [41,42,43], as well as marine invertebrates (such as sea cucumbers and sea urchin eggs) [44], and some seagrasses (e.g., *Cymodoceaceae*) [30]. Consequently, the physicochemical properties of FUCs (including molecular structure, monosaccharide composition, degree and position of sulfation, branching and MW, etc.) mainly depend on the type of source and its habitat, as well as on the season of collection and production methods [13,30,33]. FUCs isolated from the majority of brown algae are branched macromolecules whose backbone consists of alternating 1→3- and 1→4-linked α-L-fucose residues; sometimes the fucose residues are 1→2-linked [2]. However, FUCs from most echinoderms and some brown algae (e.g., *Laminariaceae* and *Chordariaceae*) are linear polysaccharides consisting of 1→3-linked α-L-fucose residues [45]. In addition, FUC can also contain other sugars, such as arabinose, glucose, xylose, rhamnose, galactose, and uronic acid, as well as acetyl groups (Figure 1) [30].

The negative charge of FUC, which allows it to form complexes with other oppositely charged molecules, is determined by the presence of sulfate groups at positions C-2 and C-4 and, sometimes, at C-3 [30,46]. FUC is readily soluble in water and insoluble in organic solvents; its solutions are not highly viscous and do not have gel-forming properties [30,47]. The viscosity of aqueous FUC solutions depends on the physicochemical properties of the biopolymer (including MW, degree of branching, and number of sulfate groups) and pH [48]. The MW of FUC ranges from 10 to 2000 kDa [30]. The precise characterization of the branching architecture of FUCs is far from complete. For example, FUC extracted from Laminaria hyperborean had a high degree of branching (22.4%), as determined by NMR. It was considered to have a comb-like structure [49]. On the other hand, the FUCs isolated from *L. japonica* were reported to have spherically symmetric structures [50]. 

The biological behavior of FUCs, as well as their technological ability to form PECs with desired properties, depends on the structural and non-structural properties of FUCs, such as their biogenic source, purity, MW and MW distribution, glycosidic linkage type and branching sites, monomer composition (fucose and sugar content), uronic acid content, degree of sulfation (sulfate content), and potential co-extracted impurities (e.g., proteins and phenolic compounds) [51]. For example, Rasin et al. [52] established the relationship between the structure of FUC and its ability to form NPs with CS (degree of deacetylation DDA ≥ 75%). It was shown that the ability to form NPs strongly depends on the structure of FUC and its MW. FUC (regular and irregular, with 340 and 123 kDa, respectively) from *Fucus evanescens*, consisting of 1→3- and 1→4-linked fucose residues, and having a 3D helical structure, formed colloidally stable NPs with CS. In contrast, the use of FUC from *Saccharina cichorioides* consisting of 1→3-linked fucose residues without 3D helical structure and high MW (773 kDa) resulted in aggregate formation.

Therefore, the comprehensive characterization of FUCs is an important step for the successful pharmaceutical R&D of FUC-based polymeric drug delivery systems with reproducible properties.

## 3. PECs as Drug Delivery Systems

Polymer-based PECs are typically spherical polymer aggregates (10 to 1000 nm) formed by non-covalent interactions (e.g., ionic or hydrogen bonds) between two different macromolecules. Polysaccharides with opposite charge (e.g., FUC and CS) usually form PECs, due to ionic interactions. PEC-based nanotechnologies are an important aspect of modern pharmaceutical R&D, as they are able to provide targeted delivery, controlled release of a drug and reduction of its therapeutic dose, thereby increasing the pharmacological efficacy of a drug and reducing its toxicity and side effects [30,53,54,55].

Interpolyelectrolyte complexation is the most commonly used method to obtain nanocarriers using FUC. The ionic nature of the negatively charged biopolymer allows it to form complexes with other oppositely charged molecules. In addition, FUC can be used, not only as an excipient for modified and targeted drug delivery, but also as a bioadjuvant and as a biological agent with its own therapeutic effects [30,35,56].

Polyelectrolyte self-assembly is commonly used to obtain FUC-based PECs [30]. The self-assembly method is a procedure to form PECs between macromolecules without introducing additional cross-linking agents [57,58]. The advantages of this technique are its simplicity and mild preparation conditions and the use of “green” solvents; moreover, this method allows to control the PEC’s parameters (e.g., size and surface charge) by mixing different weight ratios of polymers, changing the pH of the reaction mixture, or using sonication [39,59]. Furthermore, since the complex is obtained by physical cross-linking through electrostatic interactions, the toxicity associated with the use of chemical grafting reagents is eliminated [35].

Additional cross-linkers, such as genipin [60,61] and Ca^2+^ [62], are sometimes used to increase the PEC stability and modify their biopharmaceutical properties. Furthermore, another strategy in the development of core-shell polymer NPs is the method of layer-by-layer self-assembly, based on the alternating adsorption of two or more polyelectrolyte polymers on the NP surface through electrostatic interactions [63,64,65].

### 3.1. PECs Based on FUC and CS

**Figure 2:**
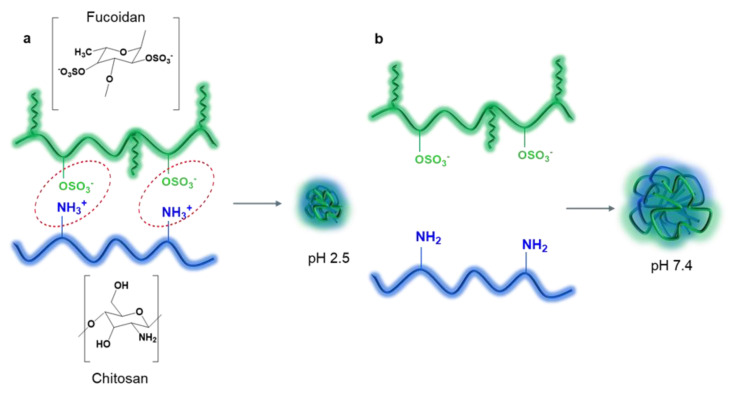
Scheme of formation of interpolyelectrolyte complexes based on FUC and CS: protonated CS electrostatically interacts with FUC at pH 2.5 (**a**); deprotonation of CS at pH 7.4 reduces electrostatic interactions with FUC and results in PEC swelling (**b**).

CS is actively used in the development of pharmaceutical formulations for improved drug delivery because it is a non-toxic, biocompatible and biodegradable polymer [66] with excellent mucoadhesive properties [66,67,68] and pronounced biological activity (including antimicrobial [69,70] and anti-inflammatory [71], as well as enhancement of paracellular transport [72,73]). The physicochemical and biological properties of CS depend on the DDA and the distribution of acetyl groups in the biopolymer chain. DDA is a crucial parameter that determines the possibility of using CS for pharmaceutical development of polymeric drug delivery systems [74]. CS is a cationic polysaccharide whose positive charge is due to the protonation of amino groups in an acidic environment; consequently, the electrostatic interaction between polyanionic (FUC) and polycationic (CS) macromolecules leads to the self-assembly of these polymers into NPs (Figure 2a) [38,59,75].

The important characteristics of CS (MW and DDA) affect both the physical properties of the resulting polymeric particles (e.g., particle size [76] and enthalpy [77]) and their biopharmaceutical parameters (permeability and drug release profile) [78,79]. For example, the enthalpy of NPs prepared from high-MW CS is higher than that of low-MW CS; and the permeability of particles based on high-MW CS is lower than that of low-MW CS [77]. Furthermore, the pH-responsive profile of FUC–CS NPs depends on the weight ratio of both polymers and their physicochemical properties [75,80].

Regarding the formation of PECs, there are various process parameters (e.g., polymer concentration and ratio, pH of the polymer solution and its ionic strength, order of mixing the components, and charge density) that can be optimized to obtain NPs with the desired size and polydispersity index (PDI) [56,62,81,82,83,84].

For example, Lee and Lim [35] investigated conditions for the formation of PECs based on CS (DDA of 75–85%, viscosity of 5–20 cP in 5% acetic acid) and FUC (*Fucus vesiculosus*) to ensure high yield, small average size (about 350 nm), and good colloidal stability. The direct working condition was used to form polymer particles: the FUC solution was added dropwise to the CS solution under continuous stirring. It was shown that PECs tended to grow at CS pH 3–3.7 (particle size was 800–900 nm), while at pH 2, 5, and 6, the particle size was 300–550 nm. The particle size was also dependent on the mass ratio of CS:FUC; with an increase in the FUC content in the system from 1:0.05 to 1:1, the particle size decreased over the entire pH range (2 to 6). As a result, a pH of 5 and a mass ratio of CS:FUC of 1:1 were suggested as specific optimum conditions for the production of PECs with the desired properties.

Oliveira et al. [56] investigated the parameters for the formation of PECs between FUC (*F. vesiculosus*, MW 45–75 kDa) and CS (MW 40–150 kDa). For this purpose, the effects of various parameters, such as the concentration of both polymers (0.25 to 1 mg/mL), the mass ratio of the polymer (CS:FUC = 1:2, 1:1, and 2:1), their pH and ionic strength (to analyze the effect of ionic strength, solutions were prepared in ultrapure water, osmotic water, and 0.9% NaCl) were evaluated. The experimental results showed that reducing the concentration of the polymer solutions from 1 mg/mL to 0.25 mg/mL resulted in a decrease in the PEC’s size from 200 to 120 nm; however, a suitable PDI (~0.16) of the resulting particles was achieved only for the polymer concentration of 0.5 mg/mL. The use of different CS:FUC mass ratios showed that an excess of FUC (1:2 ratio) resulted in the formation of particles with a size of 500 nm and a PDI of 0.6. In the case of 1:1 and 2:1 ratios, the particles formed had sizes of 120–150 nm and a PDI of 0.18 and 0.3, respectively. The pH of the solutions at which PECs with sizes below 200 nm and a PDI below 0.2 were formed was 5 for CS and 9 for FUC. The ionic strength was estimated by dissolving the polymers in different solvents of increasing ionic strength: ultrapure water, osmotic water, and 0.9% NaCl. It was shown that the particle size (120 to 350 nm) and PDI (0.18 to 0.4) increased with increasing ionic strength; optimal values of these parameters were obtained for ultrapure water. The encapsulation efficiency of an anticancer drug (gemcitabine) was 35–42%; the drug release studies showed that about 84% of the gemcitabine was released within 4 h. The cytotoxicity test showed that the gemcitabine-loaded nanosystems had an increased toxicity (about 25%), compared to the free drug, against human breast cancer cells (MDA-MB-231 cell line), without an increased toxic effect on human endothelial cells (EA.hy926 cell line).

**Figure 3:**
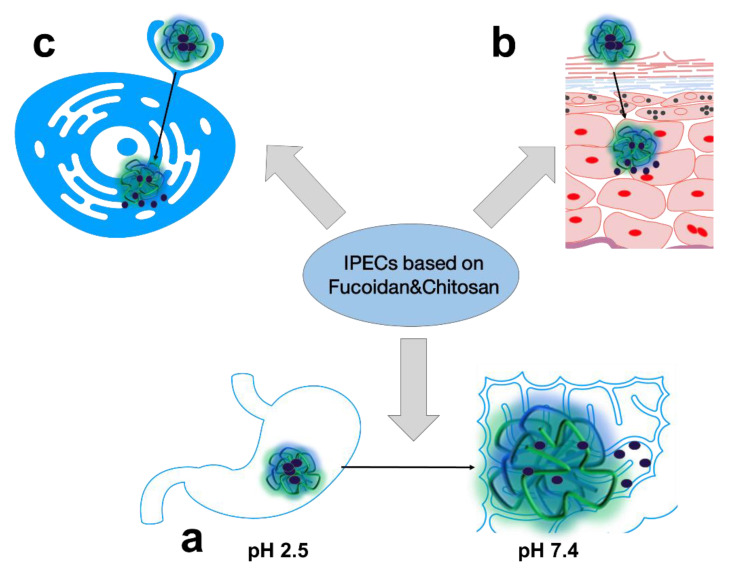
The main trends in pharmaceutical R&D of PECs based on FUC and CS: oral (**a**); topical (**b**) and targeted (**c**) drug delivery systems.

In general, the most common trends in pharmaceutical R&D of FUC- and CS-based biopolymer PECs are oral (Figure 3a), topical (Figure 3b), and targeted drug delivery (Figure 3c), which will be discussed below.

#### 3.1.1. Oral Drug Delivery Systems Based on FUC-CS Complexes

The conditions of the body environment (e.g., pH, osmolarity, various enzymes, etc.) should be taken into account in the pharmaceutical R&D of different polymeric drug delivery systems [85,86,87].

Interpolyelectrolyte interactions between the CS amino group (pKa ~ 6.5) and the FUC –OSO_3_H group (pKa 1.0–2.5 [88]) allow the formation of NPs with a pH dependent release profile [89,90]. The resulting PECs are stable at pH 2.5–3.0, due to ionic bonding between protonated amino groups of CS and ionized sulfate groups of FUC under acidic conditions (however, the NPs degrade rapidly in simulated gastric fluid at pH 2.0 if the FUC contains many carboxyl groups, which are less acidic (pKa ~ 3–4) compared to –OSO_3_H groups [91]). At physiological pH 7.4 (the pH of blood and intestinal segments where drug absorption occurs), CS amino groups are deprotonated and electrostatic interactions are weakened, causing the polymeric NPs to swell and disintegrate, releasing the loaded drug (Figure 2b). However, the pH-sensitive profile of the FUC-CS system prevents degradation under acidic gastric conditions and ensures drug absorption in the intestine, and the rate of drug release can be programmed and controlled [39]. In addition, CS increases drug residence time at the site of absorption through mucoadhesion [92], and improves intestinal permeability through enhanced paracellular drug transport mediated by the opening of a tight junction between epithelial cells [93,94]. Consequently, FUC and CS NPs have been widely used for modified oral delivery of various drugs [39,93,95].

Thus, the hard conditions of the gastrointestinal tract are a major limitation in the oral delivery of polymeric nanodrugs [96]. The pH value changes dramatically in the gastrointestinal tract. In the stomach, the pH is approximately 1.2–2.0 in the fasted state and 2.5–3.7 in the fed state. In the duodenum and jejunum, the pH rises to 6.0–6.6 and then further increases to 6.6–7.0 in the proximal ileum. In the distal ileum, and in the body fluid in the intercellular spaces between the enterocytes, the average pH is about 7.4. Therefore, the properties of the potential carrier for oral delivery should be studied at different pH values that simulate the pH of the environment in the gastrointestinal tract and in the intercellular spaces between enterocytes [93,97]. Thus, the development of a pH-sensitive carrier based on FUC–CS PECs is an important strategy for the pharmaceutical development of oral formulations. For example, Huang et al. [93] developed a pH-sensitive oral drug delivery system using CS (MW 76 kDa, DDA 72%) and FUC (*F. vesiculosus*; MW 70 kDa, total sugar and sulfate content were 30.3% and 21%, respectively). The PECs were obtained using a 1:1 polymer mass ratio. The formed particles exhibited significant pH-sensitive properties; they were quite stable at pH 2.5 (size of 380 nm and ζ-potential of +23 mV), and at pH 7.4; due to deprotonation of CS amino groups, the ζ-potential of these systems changed to −19 mV and the size increased to 1 μm. The results of the transepithelial electrical resistance test of Caco-2 cells showed that the developed polymeric nanosystems effectively enhanced the opening of the cellular tight junction.

Coutinho et al. [80] prepared polyelectrolyte NPs based on FUC (*F. vesiculosus*; MW 50–190 kDa, composed of 44.1% fucose and 26.3% sulfate, respectively) and CS (MW 50–190 kDa, DDA 75–85%) for oral methotrexate delivery. Polymeric systems with the desired properties were obtained by polyelectrolyte self-assembly methods, followed by ultrasonication; a mass ratio of FUC:CS of 5:1 was optimal at a PEC formation pH of 3. At lower pH values of 1.6 and 2.7 (simulating the fed and fasted gastric environment), the empty and methotrexate-loaded NPs exhibited stable and similar size, due to the strong polyelectrolyte interaction between the ionized amino groups of CS and the sulfate groups of FUC. When the pH is increased from 5.2 to 7.4 (simulated intestinal environment), the amino groups of CS are deprotonated, which leads to a significant swelling of the NPs, due to a decrease in the electrostatic interaction between the polymers. The sizes of empty and loaded particles increase by 1.7 and 1.4 times, respectively, indicating the stabilizing effect of methotrexate. The PDI is also increased (>0.3) for the empty NPs at pH values above 6.0, indicating an increase in the heterogeneity of the system. The size of the resulting methotrexate-loaded NPs was about 300 nm, the ζ-potential was about −30 mV, and the encapsulation and loading efficiencies were 96% and 9%, respectively. In addition, the obtained particles possessed mucoadhesive properties, which contributed to improved oral bioavailability. The prepared methotrexate delivery systems were 7 times more effective in suppressing lung cancer cell proliferation (A549 cell model) compared to the free drug.

Lee et al. [98] developed polymeric nanocarriers based on FUC (*Laminaria japonica*) and CS (MW 192 kDa; DDA ≥ 75%) to encapsulate the soluble eggshell membrane protein (SEP) to improve its bioavailability and stability in the gastrointestinal tract, and to protect it from enzymatic degradation. The obtained PECs had a spherical shape with a size of 100 nm and a positive ζ-potential of 10 mV; the loading efficiency was 80–90%. The NPs were stable in simulated gastric media (pH 1.2) and released 50% of SEP in simulated intestinal media (pH 7.4) for 10 h. The resulting polymeric carriers had good biocompatibility against Caco-2 and RAW264.7 cells, and exhibited high antioxidant activity and immunomodulatory properties.

Barboza et al. [99] developed polysaccharide NPs based on FUC (*F. vesiculosus*, MW 50–190 kDa) and CS (MW 190–310 kDa), using a polyelectrolyte self-assembly method for oral delivery of quercetin. The encapsulation efficiency and the loading efficiency were 99% and 15%, respectively. The obtained NPs had a size of 300–400 nm and a ζ-potential of about −30 mV; they retained the stability in the pH range of 2.5 to 7.4 (gastrointestinal tract conditions). The quercetin-loaded nanocarriers exhibited controlled release under simulated gastrointestinal conditions (30–40% and 55–60% of the drug were cumulatively released into fast-state simulated gastric fluid (FaSSGF, pH 1.6) and fast-state simulated intestinal fluid (FaSSIF, pH 6.5), respectively). In addition, the resulting NPs had enhanced (2-fold) antioxidant activity compared to pure quercetin at a concentration of 12.5 μg/mL.

#### 3.1.2. Topical Drug Delivery Systems Based on FUC-CS Complexes

Topical drug delivery is an effective strategy to improve pharmacotherapy by targeting the injured area and increasing the local bioavailability of drugs, thereby reducing their side effects compared to systemic drug administration, and this method is also compliant and comfortable for patients [100,101]. The main barrier to drug penetration through the skin is the outer layer of the skin, known as the stratum corneum, which consists of corneocytes. The corneocytes are located in a multilayered lipid shell (the “brick and mortar” model); overcoming this barrier is the key problem in the development of innovative drugs for transdermal delivery [102]. Recent advances in transdermal delivery involve the use of various polymeric NPs (including PECs based on mucoadhesive polymers [103]), which have great potential to improve drug penetration through the skin and can also provide controlled release of both hydrophilic and hydrophobic drugs and reduce their side effects [104,105]. Another aspect of the use of local drug delivery systems with improved properties (e.g., modified drug release, prolonged residence time, synergism and potentiation of pharmacological effect due to biologically active components of the polymer matrix, etc.) is the treatment of acute and chronic wounds [106]. 

Both CS and FUC are suitable polymeric platforms for local drug delivery, due to their beneficial properties. Costa Lima et al. [59] investigated the mechanisms of skin permeability enhancement of FUC- and CS-based NPs. FUC (*F. vesiculosus*) with MW of 50–190 kDa, CS with low MW (50–190 kDa, DDA of 75–85%) and CS with medium MW (190–310 kDa, DDA of 75–85%) were used. The FUC-CS NPs were prepared by self-assembly of oppositely charged polyelectrolytes under mild acidic conditions using ultrasound. The developed particles had average sizes of 288 and 305 nm and negative ζ-potentials of −46 and −39 mV for NPs based on low and medium MW CS, respectively. The obtained particles had pH-responsive properties; the size of polymeric particles based on medium MW CS did not change practically with pH (pH was 3.0, 6.0, and 7.4). However, the low-FUC MW CS particles swelled strongly with increasing pH, and their size increased from 288 and 342 to 1711 nm for the three pH conditions of 3.0, 6.0, and 7.4, apparently due to insufficient positive charges to maintain the colloidal stability of the NPs. Using synchrotron-based Fourier transform infrared microspectroscopy, it was shown that FUC-CS NPs obtained with low MW CS improved the permeability of calcein through the skin by disorganizing the lipids in the stratum corneum layer. The NPs obtained with medium MW CS changed the lipid structure to a more ordered and dense conformation, which did not affect the diffusion of calcein.

Barbosa et al. [75] developed PECs of FUC (*F. vesiculosus*, MW 50–190 kDa) and CS (MW 190–310 kDa), based on their electrostatic interactions with enhanced skin permeability for local delivery of methotrexate. The obtained polymeric particles had a loading efficiency of about 14% and an encapsulation efficiency of 80–96%; the NP size was 300–500 nm, ζ-potential was both positive (+60 mV) and negative (−40 and −45 mV), depending on the mass ratio of FUC:CS, respectively). Encapsulated methotrexate had reduced cytotoxicity compared to free methotrexate against L929 fibroblasts and HaCaT keratinocyte cell lines. Skin permeability studies (porcine ear skin model) showed that the permeability of negatively charged particles was 2.7- and 3.3-fold higher compared to free methotrexate.

Rao et al. [107] developed an efficient polymer system based on a PECs of CS and FUC for platelet-rich plasma (PRP) encapsulation. The PECs were obtained by simple stirring at room temperature by droplet addition of a CS solution containing PRP to a FUC solution (95% encapsulation efficiency). The resulting CS–FUC colloids had a spherical shape and provided a prolonged release of PRP for 72 h. Biological tests showed that the obtained systems were biocompatible and enhanced fibroblast and keratinocyte proliferation by increasing protein Ki67 expression and fibroblast migration.

Huang et al. [81] developed biodegradable NPs based on CS (MW 645 kDa; DDA > 75%) and FUC (*F. vesiculosus*, MW 80 kDa) to deliver basic fibroblast growth factor (bFGF) for neural tissue engineering. At CS:FUC weight ratios of 1:1, 1:5, and 1:10 (charge ratios of 1.66:1, 1.33:1, and 1.16:1), PECs with sizes of 170, 280, and 250 nm and ζ-potentials of +25, −52, and −53 mV were formed. Encapsulation efficiency was approximately 90% for all three formulations. The cumulative release of bFGF from the polymeric NPs was 25 times slower compared to the free drug. In vitro studies showed that the use of polymeric delivery systems significantly reduced the amount of bFGF required for neurite outgrowth.

#### 3.1.3. Targeted Drug Delivery Systems Based on FUC-CS PECs

Targeted drug delivery to the specific organ and intracellular uptake provide better systemic and local therapeutic effects and significantly reduce toxicity. Due to the enhanced permeation and retention (EPR) effect, NPs with a hydrodynamic diameter of approximately 10–400 nm can target desired sites through passive targeting pathways [108,109,110]. In addition, various biologically active molecules can provide specific targeting by binding to specific receptors expressed at the site of injury (e.g., strong affinity of FUC and hyaluronan for P-selectin and CD44 receptors, respectively) [111,112,113]. P-selectin is overexpressed in many types of cancer (including bladder, prostate, lung, and breast cancer [27,114,115]) and is important for metastasis by promoting cancer cell adhesion to the endothelium and platelet activation in distant organs [12,65,112]. CD44 is a suitable receptor for active targeting, due to its overexpression in various inflammatory sites (including cancer cells). The affinity of nanocarriers for CD44 can be increased by conjugation with hyaluronan, allowing for enhanced cellular uptake through the hyaluronan-CD44 receptor-mediated endocytosis pathway [116,117,118]. In addition, targeted drug delivery to macrophages, which can be host cells for microbes, is a promising way to treat various bacterial infections [119]. 

The polysaccharides CS and FUC have been widely used for targeted nanoformulations, due to their advantages, such as low toxicity, hydrophobic properties, complementary mucociliary clearance, and mucoadhesive and biological activity; in addition, FUC has affinity for various cells and receptors (such as macrophages, chemokines, growth factors, and P-selectin), which enhances drug delivery to the target site [120,121,122,123,124]. For example, Ho et al. [125] developed macrophage-targeted NPs consisting of polyethylene glycol-gelatin conjugates coated with an anionic polysaccharide mixture of hyaluronan (MW 200 kDa) and FUC (*F. vesiculosus*, MW 20–200 kDa) to encapsulate and deliver the anti-inflammatory agent epigallocatechin-3-gallate (EGCG). The developed NPs had an average size of 217 nm, a ζ-potential of −34 mV and high EGCG loading efficiency (52%). Hyaluronan and FUC molecules also exhibited biological activity (targeting CD44 receptors and P-selectin, and suppressing cell migration in macrophages, respectively).

Don et al. [126] formed multitargeting (pH- and P-selectin-responsive) polymeric nanocarriers based on CS (MW 61 kDa; DDA 91%) and FUC (*L. japonica*) to load hydrophobic curcumin. For this purpose, an alcoholic solution of curcumin was homogeneously mixed with a solution of FUC, and then the resulting mixture was slowly added to an acidic solution of CS while stirring; the encapsulation efficiency was 46 to 88% and the drug loading efficiency was 6 to 10%. The formed particles had an average hydrodynamic size of about 170 nm, and a positive ζ-potential of 25 mV, due to a 1.4–1.8-fold mass excess of CS relative to FUC. The developed curcumin delivery systems had a pronounced anti-inflammatory effect and improved delivery, distribution, and accumulation of the drug at inflammatory brain sites during intranasal delivery.

Liu et al. [127] obtained PECs based on FUC (*F. vesiculosus*) and CS (DDA ≥ 75%) with antioxidant and anti-inflammatory properties for the treatment of atherosclerosis. The NPs were obtained by polycation-polyanion self-assembly under ultrasound conditions (a solution of FUC pH 9 was added to an acetic acid solution of CS pH 5). Optimal parameters for the formation of PECs were found: polymer solution concentrations in the range of 0.75–1.5 mg/mL and a CS:FUC ratio of 3:1. The developed polymer particles had a size of 152 nm and a ζ-potential of 25 mV at pH 7.4. The obtained NPs effectively inhibited local oxidative stress and inflammation by acting on P-selectin in atheromatous plaques, thereby preventing atherosclerosis. This effect was confirmed in an in vivo experiment by monitoring atherosclerotic plaques with CFNs in ApoE−/− mice.

Wu et al. [128] obtained PECs with antioxidant activity (reduction of cellular oxidative damage), based on oligo-FUC (*L. japonica*, MW 500–1500 Da) and CS (MW 50–190 kDa, DDA 75–85%) by self-assembly of two oppositely charged polyelectrolyte biopolymers. The developed NPs had hydrodynamic diameters from 190 to 230 nm and a neutral ζ-potential (−3.2 mV at pH 2.5 and −1.5 mV at pH 7.4). The obtained systems exhibited radioprotective properties in mice irradiated at 5 Gy; the NPs prevented radiation-induced lipid peroxidation and restored the enzymatic and non-enzymatic status of antioxidants in the intestine.

Huang et al. [58] proposed a nanoantibiotic based on CS (MW 38 kDa, DDA ≥ 75%) and FUC (*F. vesiculosus*, MW 10–55 kDa, monosaccharide composition: fucose (1849. 6 μmol/g), mannose (149.6 μmol/g), xylose (239.5 μmol/g), and galactose (122.3 μmol/g)) for targeted pulmonary delivery of gentamicin with biphasic release. Optimal polymer particles (size 300–400 nm, ζ-potential +40 mV, encapsulation efficiency 90–95%) were formed at 3–4 times the mass excess of CS. The developed delivery system showed zero order gentamicin release for the first 10 h (70% of the drug); the release then continued for 72 h and reached a value of 99%. Encapsulation of the antibiotic improved its pharmacokinetic properties (the AUC:MIC ratio during intravenous administration for encapsulated and free gentamicin was 19.94 and 0.90, respectively).

Elbi et al. [90] developed ciprofloxacin-loaded CS (MW 100–150 kDa, DDA ~ 85%) NPs and then coated them with FUC (MW 20–200 kDa) to enhance the penetration of the antibiotic into the intracellular compartments of macrophages. The obtained PECs have sizes of 124 and 320 nm, and ζ-potentials of +41.4 and −47 mV, respectively. The release profile of ciprofloxacin (citrate buffer, pH 5.0) showed an initial burst release (51% on day 1), and then the release reached 93% on day 14. The FUC coating prevented the initial burst release; the release was 26% and 89% of ciprofloxacin on day 1 and day 14, respectively. The intracellular anti-Salmonella activity of FUC-coated polymer systems was 2-fold higher than FUC-free NPs and 6-fold higher than free ciprofloxacin.

Huang et al. [129] prepared PECs based on CS (MM 38 kDa, DDA ≥ 75%) and FUC (*F. vesiculosus*) in a 5:1 weight ratio; the particles had a diameter of 230–250 nm. In vitro tests showed that the developed systems controlled the release of the model drug gentamicin for 72 h with 99% release, and also showed a good antioxidant effect by absorbing 1,1-diphenyl-2-picrylhydrazyl (DPPH), reducing the concentration of intracellular reactive oxygen species (ROS) and superoxide anion (O_2_^−^) in stimulated macrophages. 

Choi et al. [130] developed an intracellular delivery system for the hydrophobic pro-oxidant piperlongumine based on FUC and CS at pH 5.0. Piperlongumine is a pro-oxidant agent whose mechanism of action is based on inducing cancer-specific apoptosis by increasing oxidative stress in cancer cells. Therefore, the obtained piperlongumine-loaded polymer NPs killed human prostate cancer cells via piperlongumine-induced intracellular reactive oxygen species generation. Protonated CS in the acidic state can be efficiently complexed with negatively charged FUC. The resulting NPs with an average size of 215 nm and a positive ζ-potential (19 mV) effectively encapsulated piperlongumine (encapsulation efficiency was 16%), increasing its water solubility and bioavailability. The release profile of piperlongumine in phosphate-buffered saline (pH 7.4) was 80% for 24 h. The developed NPs based on CS and FUC effectively killed cancer cells (1.5 times) in human prostate cancer cell model (PC-3) compared to the free drug.

In addition to CS, other polycationic polymers can also be used to form FUC-based PECs for targeted drug delivery. For example, Zhang et al. [131] used a polyanionic FUC (from *Macrocystis pyrifera*) and a polycationic polylysine to coat lipid particles loaded with doxorubicin in layers for P-selectin-mediated targeted delivery. The resulting particles had a hydrodynamic size of 200 nm and a ζ-potential of −47 mV. Biodistribution studies showed increased tumor uptake of the drug (4.4-fold higher than control), due to the affinity of FUC for P-selectin and the enhanced EPR effect.

### 3.2. PECs Based on FUC with CS Derivatives

CS is a polymer that dissolves in acidic conditions, due to the protonation of amino groups. At pH values above 6.5, CS is deprotonated, which decreases its solubility, mucoadhesion, biological activity and ability to open a tight junction between cells [132]. Modification of CS to obtain its water-soluble derivatives has great potential for the development of PECs for modified drug delivery. For example, grafting arginine (pKa ~ 12.5) to the amino group of CS [61,133] increases solubility in neutral medium and increases paracellular permeability, since substituted arginine retains positive charge at neutral and basic pH levels [95,134]. In addition, various quaternized cationic substituents increase the positive charge density of the macromolecule and, thus, influence the size and charge of the formed PECs, on which the biopharmaceutical properties of these systems (encapsulation and drug loading efficiency, release profile, etc.) depend [7,132].

Huang et al. [135] obtained polymeric epigallocatechin gallate (EGCG) delivery systems based on FUC (*F. vesiculosus*; MW 58 kDa, 44% fucose and 26% sulfate content, respectively) and a water-soluble cationic quaternary CS (MW 35 kDa; DDA 85%) derivative with glycidyl trimethylammonium chloride (GTMAC) substituent. The polymeric systems were prepared by electrostatic interaction between the negatively charged sulfate groups of FUC and the protonated amino groups of CS and GTMAC moieties. The NPs obtained had a hydrodynamic size of about 200 nm and a positive ζ-potential of about 30 mV. Encapsulation of EGCG in polymeric nanocarriers prolonged drug release, both under conditions simulating the gastric environment (pH 2.0), and in the model environment of the small intestine (pH 6.8); the release was 20–30% and 40–45%, respectively. The resulting systems enhanced the transepithelial permeability of EGCG in the Caco-2 intestinal absorption model. In addition, the polysaccharides used acted as adjuvants; FUC, in combination with EGCG, increased the enzyme inhibitory activity against α-amylase (by 2.82–4.92-fold) and α-glucosidase (by 1.35–1.67-fold), and quaternary CS enhanced the antibacterial effect of EGCG against Staphylococcus aureus (about 5-fold) and Escherichia coli (about 8-fold).

Tsai et al. [136] and Chuang et al. [137] obtained FUC (*F. vesiculosus*) and trimethylchitosan-based NPs with enhanced immunostimulatory efficacy of anthrax vaccine adsorbed (AVA). The original CS had a viscosity of 3.6 mPa·s (5 g/L)) and a DDA of 93.8%. The resulting particles with a positive (+21 mV) or negative (−32 mV) ζ-potential and an average size of 230 and 220 nm, respectively, were obtained by complexing differentially charged polyelectrolytes. Both types of charged NPs showed no cytotoxicity on L929, A549 and JAWS II dendritic cells. In vivo studies in an A/J mouse model showed that AVA-loaded CS-FUC polymeric particles induced an effective immune response with high IgG titers, and provided 100% protection during *Bacillus anthracis* spore infection. In addition, analysis of specific IgG1 and IgG2a confirmed that the developed systems significantly stimulated humoral immunity.

Tsai et al. [132] developed oral insulin delivery systems based on FUC (*F. vesiculosus*, MW 31.7 kDa and 26.5% sulfate content) and trimethylchitosan (synthesized from CS with MW 35 kDa and DDA 85%) using a simple polyelectrolyte complex method. The size of the obtained particles was 250–260 nm in the pH range of 2–6.5, which increased to 450 nm at pH 7.4. The ζ-potential of the PECs gradually decreased from 37 to 13 mV when the pH was increased from 2 to 7.4. The developed systems exhibited pH-dependent modified release; the cumulative insulin release was 20 and 30% within 2 h, at pH 2 and 6.8, respectively, and then the release reached 70% within 12 h. Intestinal permeability studies through the Caco-2 cell monolayer showed increased absorption of encapsulated insulin compared with native insulin (permeability coefficient (Paap) values were 7.39 × 10^−6^ cm/s and below 1 × 10^−6^ cm/s, respectively). Moreover, the obtained NPs showed α-glucosidase inhibitory activity (inhibition ratio was 33.2% at a concentration of 2 mg/mL).

Huang et al. [62] developed pH-sensitive NPs based on O-carboxymethyl chitosan (synthesized from CS with MW 192 kDa and DDA ≥ 75%) and FUC (*F. vesiculosus*) for oral curcumin delivery by ionic cross-linking with calcium ions. With a weight ratio of polysaccharides of 1:1, the developed NPs had a size of 270 nm, a ζ-potential of −22 mV, and an encapsulation efficiency of 93%, and also exhibited pH-sensitive properties (encapsulated curcumin was stable at pH 2.5 for 2 h; however, 80% of the drug was released within 7 h in a simulated intestinal environment (pH 7.4). An in vitro Caco-2 model of cellular uptake showed that the developed NPs were internalized by cells through energy-dependent endocytic pathways.

### 3.3. Delivery Systems of Hydrophobic Drugs

An important challenge in the pharmaceutical development of hydrophobic drug delivery systems is to increase their solubility. Increasing the hydrophilicity of the drug improves its pharmaceutical solubility, biodistribution and bioavailability, resulting in greater therapeutic efficacy of the drug [108]. There are several ways to increase drug solubility that do not modify the molecule and do not alter its pharmacological active sites, such as incorporating a poorly water-soluble compound into PECs (for example, the hydrophobic nature of the acetyl group of CS [74] allows loading some hydrophobic drugs into PECs based on CS and its derivatives [138]), into oil/water microemulsions, or into hydrophobic polymeric particles that are further coated with a shell of water-soluble polymers (e.g., CS, and FUC), as well as obtaining dendrimer-based clusters [139,140,141,142]. 

**Figure 4:**
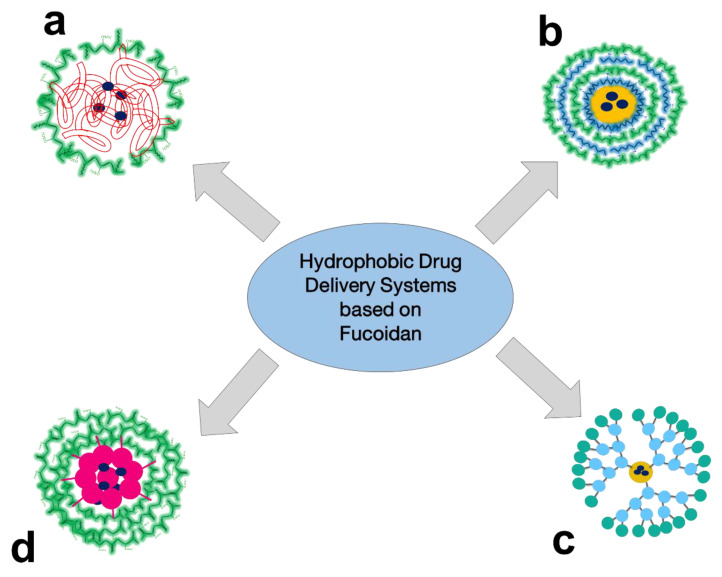
The main techniques for pharmaceutical R&D of FUC-based PECs for delivery of hydrophobic drugs: NP with hydrophobic core and hydrophilic shell (**a**), NP with hydrophobic core and layer-by-layer shell (**b**), dendrimer-based NP (**c**), and self-assembled NP based on hydrophobic-modified FUC (**d**).

In general, the key techniques for pharmaceutical R&D of FUC-based PECs for hydrophobic drug delivery are obtaining NPs with a hydrophobic core and hydrophilic shell (Figure 4a), including a layer-by-layer shell (Figure 4b), dendrimer-based NPs (Figure 4c), and self-assembled NPs based on hydrophobically modified FUC (Figure 4d). The different techniques for obtaining FUC-based hydrophobic drug delivery systems are reviewed below.

Lee et al. [143] developed a curcumin delivery system to improve its solubility, stability, and bioavailability during oral administration. For this purpose, curcumin was incorporated into an oil/water emulsion by dissolving it in soybean oil with Tween 80 as an emulsifier, and then the surface of the resulting oil droplets was coated with a pH-sensitive PEC based on CS and FUC. A maximum curcumin loading efficiency of 35% was achieved for formulations with a mass ratio of FUC:CS of 1:1, prepared using a CS solution with pH of 3.7 and 6.0. The size of the obtained particles was little dependent on pH, remaining at 200 to 250 nm when the pH was changed from 2.0 to 6.0. However, the ζ-potential of these systems was pH-sensitive, at pH 3–3.7 the maximum ζ-potential of the particles was observed (15–20 mV), and at pH 2, 5, and 6 the ζ-potential was 11, 6, and 0 mV, respectively. The developed nanocarriers prolonged the release of curcumin for 55 h.

Lai et al. [140] developed a delivery system (hydrophobic core-hydrophilic shell) for the encapsulation of the hydrophobic anticancer drug docetaxel based on FUC (*F. vesiculosus*, MW 20–200 kDa, 27.0% sulfate content) and poly(lactide-co-glycolide) (PLGA). The polymeric carriers were prepared by mixing and emulsifying an aqueous solution of FUC with a chloroform solution of PLGA—docetaxel, followed by removal of the chloroform. The encapsulation efficiency was 45 to 80%, and the loading efficiency was 7 to 34%, depending on the PLGA content. The developed particles had hydrodynamic sizes of 200 to 500 nm, and a negative ζ-potential of about −60 mV; they were also characterized by delayed release of docetaxel (10–30% within 70 h). In addition, the resulting docetaxel delivery systems showed improved anticancer activity in the MDA-MB-231 breast cancer cell model compared to the free drug.

Wang et al. [144] developed a novel methotrexate delivery system by a simple polyelectrolyte self-assembly method, using polyallylamine hydrochloride (MW 120–200 kDa) and FUC (MW 200–400 kDa) as the polycation and polyanion, respectively. The resulting systems had a loading efficiency of 13%, a hydrodynamic size of 163 nm, and a ζ-potential of approximately −30 mV. In vitro drug release studies at pH 6.0 (simulating the acidic environment of a tumor) showed that the release of methotrexate was prolonged, with 80% of the drug released within 240 h. The developed NPs showed anticancer activity on MCF-7 and HeLa cells.

Pawar et al. [145] prepared doxorubicin-loaded nanocontainers using the immunomodulatory polysaccharide FUC (*F. vesiculosus*) and polyethyleneimine (MW 25 kDa). The NPs were electrostatically assembled by intermolecular electrostatic interactions of a polyanionic FUC with a polycationic polyethyleneimine; the encapsulation efficiency ranged from 35 to 82%. The resulting PECs had hydrodynamic diameters of 40–85 nm and ζ-potentials of −34 to −42 mV. NPs exhibited enhanced cytotoxicity (2.64-fold), cell cycle arrest in the G1-S phase (34.65%), and apoptosis (66.12%) in tumor cells compared to free doxorubicin. In vivo anticancer activity in BALB/c mice with 4T1-induced tumors demonstrated improved efficacy (approximately 3-fold) of encapsulated doxorubicin compared to the free drug. In addition, a pharmacokinetic study showed that administration of the engineered polymer NPs to tumor-infected mice induced a biphasic change in serum doxorubicin levels with peaks at 1 and 6 h, thus maintaining the preferential localization of the chemotherapeutic agent in the tumor.

#### 3.3.1. Dendrimer Drug Delivery Systems

Hierarchical polyionic complex dendrimer vesicles exhibit “host-guest” chemical properties, because the hydrophilic functional groups on the outer surface enhance their solubility in water, while the inner core and branching enhance the solubility of hydrophobic drugs; in addition, dendrimers increase the stability of NPs and clusters [142,146]. However, unmodified polyamidoamine (PAMAM) dendrimers have no specific affinity for cells and receptors, and a high positive charge can increase their cytotoxicity. Therefore, it is reasonable to coat them with various suitable polyanionic polymers, such as FUC [146,147].

Chung et al. [146] developed a biocompatible theranostic nanoplatform based on the co-assembly of a nanocomplex of functional polysaccharide FUC (*L. japonica*, MW 8775 Da, 22% L-fucose and 34% sulfate, respectively) and polyamidoamine (PAMAM) dendrimer, doped with MnO_2_ NPs for targeted delivery of the photosensitizer verteporfin. Verteporfin inhibits tumor growth by suppressing the Hippo-YAP (yes-associated protein) signaling cascade. YAP is known to regulate the epidermal growth factor receptor in lung cancer cells and to induce glycolysis in colon cancer cells [148,149]; moreover, YAP overexpression correlates with cancer stemness and negative prognosis in triple-negative breast cancer. The developed dendrimer-FUC polyion nanocomplex had a size of 150–200 nm and a negative ζ-potential of about −30 mV. The fabricated nanoclusters exhibited redox-reactive verteporfin release; 30% of the drug was rapidly released within 10 min in the presence of the disulfide reducing agent dithiothreitol (DTT), and then completely released within 120 min, whereas verteporfin was not released in the DTT-free medium. The resulting nanocomplex reduced tumor hypoxia, suppressed oncogenic signaling, and reduced immunosuppression, which improved therapeutic efficacy and enhanced anti-tumor immunity.

#### 3.3.2. PECs Based on FUC and Proteins/Polypeptides

The incorporation of biologically active substances with low water solubility and poor chemical stability into pharmaceutical formulations is extremely challenging [150,151,152,153].

**Figure 5:**
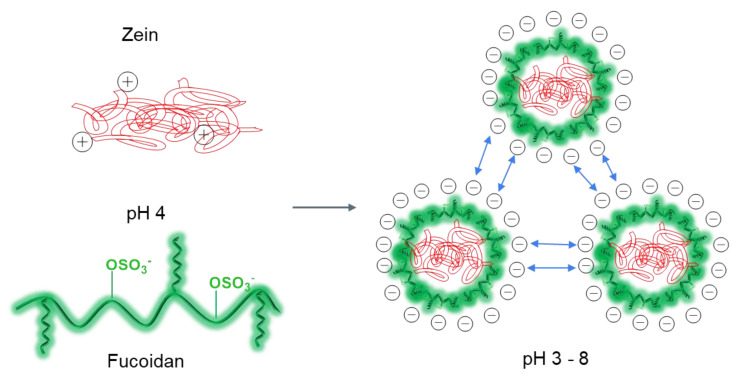
Scheme of formation of interpolymer complexes based on FUC macromolecules and zein NPs: ionic interaction of two oppositely charged polymers leads to the formation of systems stable in the pH range of 3–8, due to electrostatic repulsion of negative charges on the surface of zein NPs.

Proteins with a high content of non-polar amino acids (e.g., zein) are readily soluble in ethanol/water systems (55–95%), strong alkaline (pH > 11) and anionic surfactant solutions [151,154]. Zein consists of α-, β-, γ-, and δ-zein, the proteins with different MWs and extraction modes. Zein has hydrophobic and hydrophilic domains, but is often considered a hydrophobic protein, due to its insolubility in water. Amino acid composition analysis shows that zein has a high proportion (>50%) of non-polar amino acids (leucine, proline, alanine, and phenylalanine), which makes zein insoluble in water but soluble in 60–90% ethanol solution. Momany et. al. proposed an α-zein structure consisting of 9 to 10 antiparallel helical segments connected by glutamine-rich turns [155]. Small angle X-ray scattering showed that reduced α-zeins exist as asymmetric particles, with a length of about 13 nm in 70% aqueous ethanol [156]. The top and bottom of zein are hydrophilic, while its outer surface is hydrophobic, making it amphiphilic. This model explains the amphiphilic and self-assembling nature of zein. When dissolved in an aqueous ethanol solution, zein molecules are organized into colloidal NPs by anti-solvent precipitation (dropwise addition to a large water bath) or by precipitation from a strongly alkaline solution when it is acidified to neutral pH. However, such zein NPs are unstable (sensitive to salt concentration and pH changes and aggregate near the zein isoelectric point) and prone to aggregation, so they are usually stabilized with additional biopolymer materials, such as polysaccharides (e.g., FUC, chondroitin sulfate, pectin, and alginate) [151,157,158,159] and proteins (e.g., sodium caseinate, and serum protein isolate) [160,161]. The pI of zein is 6.2 [162]. FUCs are strong polyelectrolytes with pK_a_ between 1.0–2.5 [88], so the electrostatic interactions of FUC with zein are possible in acidic solutions at pK_a_ < pH < pI. Therefore, the zein molecule is positively charged at pH 4.0 (ζ-potential of +22 mV) and is able to interact with the negatively charged FUC polyanion (ζ-potential of about −44 mV at pH 4.0) to form colloidal stable composite NPs, due to electrostatic attraction (Figure 5) [163].

Zhang et al. [36] obtained composite of zein and FUC (*L. japonica*, viscosity 3–15 cP, MW 50–200 kDa, sulfate content 25.5%, L-fucose 24.6%, other monosaccharide 7%) NPs by the antisolvent precipitation method for encapsulation and delivery of hydrophobic curcumin (with an encapsulation efficiency of 81%). The resulting NPs had stable sizes (about 150 nm) at pH 3.0–8.0, due to stabilization by the sulfate group of FUC. The study of the effect of salt concentration showed that, at pH 4 and 7, the particles remained stable when the concentration of sodium chloride was increased to 20 mM, but the particle size slightly increased (300 and 280 nm, respectively), and the PDI remained low (0.1–0.2). The developed polymeric nanocarriers had a modified release of curcumin in the gastrointestinal tract. The systems exhibited sustained release in simulated gastric fluid (SGF; 2.0 mg/mL NaCl and 3.2 mg/mL pepsin, pH 2.0), and curcumin release was approximately 10% in 90 min. In simulated intestinal fluid (SIF; 0.8 mg/mL pancreatin, 6.8 mg/mL K2HPO4, 12 mg/mL bile extract, and 8.8 mg/mL NaCl, pH 7.4), a significant burst of drug release was observed and, after 30 min, curcumin release (62% of drug) reached a plateau.

Liu et al. [163] developed delivery systems for the water-soluble biologically active substance resveratrol based on FUC (*L. japonica*, MW 120 kDa) and zein PECs. The obtained NPs, with an average size of about 120 nm, had a spherical shape and a ζ-potential of −41 mV; the encapsulation efficiency of resveratrol was 95%. The developed NPs had high colloidal stability; they showed controlled release of resveratrol, and extremely low cytotoxicity to HIEC-6 cells.

Zhang et al. [164] obtained quercetin-loaded NPs based on zein and FUC (*L. japonica*, viscosity 3–15 cP, MW 50–200 kDa) by an antisolvent precipitation. The zein–FUC NPs were formed via the electrostatic interactions, hydrogen bonding, and hydrophobic interactions. The addition of Ca^2+^ ions enhanced the release of bioactive substances from the engineered NPs in simulated digestive media.

Liu et al. [158] obtained core-shell NPs based on FUC (*F. vesiculosus*) and zein for enhanced delivery of pterostilbene. The initial zein NPs had a hydrodynamic size of 80 nm and a positive ζ-potential of +50 mV. After coating the zein surface with FUC, NPs with a size of 120–140 nm and a negative ζ-potential of about −40 mV were obtained. The encapsulation efficiency was 80–95%, and the loading efficiency was 3 to 8%. The in vitro release profile of pterostilbene from the zein-FUC NPs was investigated in fed state simulated gastric fluid (FeSSGF) and under simulated intestinal fluid (SIF) conditions. The developed NPs exhibited a delayed release of pterostilbene in FeSSGF medium; after 120 min, the cumulative amount of pterostilbene was 20%. A rapid drug release was then observed in SIF medium for the first 60 min. The cytotoxicity test showed that the zein-FUC NPs were non-toxic to Caco-2, HK-2, and L-02 cells.

Liu et al. [151] developed zein-caseinate-FUC (*L. japonica*, MW 120 kDa) NPs to encapsulate the hydrophobic phytochemical pterostilbene (a dimethylated analog of resveratrol). The resulting NPs were formed, due to electrostatic, hydrophobic, and hydrogen interactions; they had a globular microstructure, their average size was about 62 nm, the ζ potential was −29.1 mV, and the pterostilbene encapsulation efficiency was 95.2%. The NPs had excellent colloidal stability over a wide pH range (2.0–8.0), simulating gastrointestinal conditions. In addition, the prepared NPs exhibited controlled release of pterostilbene in vitro (60–80% in 6 h) and were biocompatible and non-cytotoxic.

Etman et al. [165] obtained quinacrine-containing NPs based on FUC (from *Undaria pinnatifida*, MW 50–100 kDa) and lactoferrin by self-assembly of two oppositely charged polyelectrolytes. The formed particles had a size of about 200 nm, an encapsulation efficiency of 80%, and a pH-dependent sustained release of the drug in an acidic tumor environment (almost 30% release after 2 h). Biological experiments showed that the anticancer activity of quinacrine loaded into NPs based on FUC and lactoferrin was 5.7-fold higher than that of the free drug. The developed NPs showed a 68% reduction in tumor volume in in vivo tests compared to 20% for free quinacrine.

Fan et al. [166] developed a protein-polysaccharide complex through electrostatic interaction between soy protein isolate (Spi) and FUC to efficiently load lipid-soluble curcumin. Stable in the pH range of 2 to 9 and at ionic strength less than 100 mM, PECs were obtained at a polymer mass ratio of 1:1, had a spherical core-shell structure, a size of about 237 nm, PDI of 0.15, and ζ-potential of about −20 mV. The curcumin loading efficiency was >95%. The system had a modified drug release; the particles were more stable in simulated gastric fluid (+pepsin), 40% of the curcumin was released in 1.5 h; in simulated intestinal fluid (+pancreatin), 80–90% of the drug was released in 3 h.

#### 3.3.3. PECs Based on FUC and Arginine-Containing Proteins

As mentioned above, PECs of FUC with CS are promising oral delivery systems, due to their pH sensitivity. However, these PECs become unstable under normal physiological conditions (pH 7.4), due to deprotonation of CS amino groups at pH above 6.5. Therefore, the development of nanoplatforms for intravenous drug delivery based on FUC and CS is limited, due to their rapid degradation in the circulation [82]. In this regard, the use of various proteins and peptides containing arginine (pKa 12.5) is a suitable strategy for the development of polymeric nanodrugs that are stable at physiological pH [82,167]. For example, protamine is a strongly basic protein, consisting of more than 60% arginine; protamine penetrates the membrane, due to its strong positive charge [168]. Lowering the pH in the tumor cell to 4.5–5.5 can induce a conformational change in protamine, resulting in charge conversion and an increase in particle size; in addition, some carcinomas express protein-digesting enzymes [169].

Lu et al. [82] developed pH/enzyme-reactive PECs based on FUC (*L. japonica*, 34% sulfate, MW 80 kDa) and cationic protamine polypeptide (MM 5 kDa) for P-selectin-mediated targeted delivery of the anticancer drug doxorubicin. Addition of protamine solution to FUC solution (the normal operating condition) resulted in the formation of particles whose hydrodynamic size varied 150, 400, and 250 nm with increasing amounts of protamine. The reverse addition of FUC solution to protamine solution (the reverse operating condition) resulted in the formation of FUC–protamine particles whose hydrodynamic diameters increased smoothly (from 70 to 250 nm) as the weight ratio of protamine to FUC increased. The FUC–protamine and protamine–FUC PECs had negative ζ-potentials (−16 to −41 mV) and (−22 to −43 mV), respectively. For both mixing methods, the resulting colloidal nanosuspensions were stable only at a protamine:FUC weight ratio below 0.9. Release profile studies showed that doxorubicin release was significantly accelerated when the pH of the medium was reduced from 7.4 to 4.5 (late endosome and cancer cell lysosome model): 78.3% of doxorubicin was released after 12 h and up to 93.1% of doxorubicin after 24 h. In addition, the developed systems showed an improved inhibitory effect against the metastatic breast cancer cell line (MDA-MB-231).

Hsiao et al. [167] developed a targeted delivery system for doxorubicin based on FUC and arginine-modified gelatin; the desired NPs were prepared by electrostatic interactions between two oppositely charged biopolymers. The optimal FUC:arginine-gelatin ratio was 2:1, and the optimized FUC concentrations ranged from 0.8 to 1.2 mg/mL (resulting in PECs of 230–300 nm with PDI of 0.2 and negative ζ-potential of −(34–36) mV). The developed NPs exhibited high drug-loading efficiency (88–97%) and pH-sensitive drug release. At pH 5.0, 20 and 40% of doxorubicin were released in the same time intervals. The results of in vitro and in vivo experiments showed that the FUC-containing systems targeted P-selectin expressed by cancer cells and allowed the drug to accumulate at the tumor site.

## 4. Modification Methods to Improve Drug Delivery

### 4.1. PEC Modification of the NP Surface. Layer-by-Layer Self-Assembly Techniques

Surface modification is an attractive way to improve the biopharmaceutical properties of NPs (including prolonged drug residence time due to coating with mucoadhesive polymers, targeted delivery due to specific binding to target cells, modified drug release, etc.) as well as the biocompatibility of polymeric nanocarriers [170]. Previously, we reviewed the coating of zein NPs with FUC to improve their stability, targeting and biopharmaceutical properties (Figure 5).

**Figure 6:**
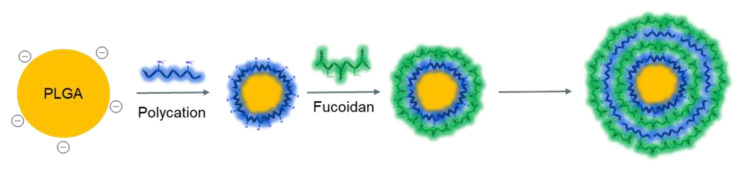
Scheme of the formation of core-shell polycomplexes by modifying the surface of NPs through successive deposition of counter-polyions.

Among the various methods of surface modification of biomaterials, the layer-by-layer self-assembly of polymers is also widely used for the formation of various supramolecular structures, due to its simple and “green” procedure. An important advantage of the layer-by-layer self-assembly method is the possibility to obtain polymeric core-shell nanostructures, in which substances of different natures (including hydrophobic drugs, to improve their hydrophilicity and bioavailability) can be loaded, and the multilayering allows to program and control the drug release profile [64]. Successful formation of polyelectrolyte multilayers can be achieved by successive deposition of different polycations (e.g., CS, poly-L-ornithine, etc.) and polyanions (including FUC) (Figure 6) [64,171,172].

For example, Wang et al. [173] proposed a drug delivery system containing calcium carbonate microparticles as a core, coated with a shell of alternatively charged polyelectrolytes (poly-L-ornithine and FUC), using the layer-by-layer self-assembly process. The average particle size obtained after deposition of polymer multilayers increased from 1.91 to 2.03 μm, and the doxorubicin encapsulation efficiency was 70%. The developed systems were characterized by a prolonged drug release profile (35% doxorubicin in 150 h); this controlled release resulted in significant antiproliferative efficacy of encapsulated doxorubicin in a breast cancer model (MCF-7 cell line).

Cai et al. [63] used layer-by-layer self-assembly to obtain NPs based on poly(lactide-co-glycolide) (PLGA), poly-L-ornithine, and FUC. Polycationic poly-L-ornithine and polyanionic FUC were deposited layer-by-layer on the surface of PLGA particles (core) by electrostatic interaction; the size of the obtained particles was about 170 nm. In vitro (mammary epithelial cells (MCF-10A cell line)) and in vivo (SPF mouse model) tests showed that the developed potential drug delivery systems were biocompatible and non-toxic.

Fan et al. [64] obtained a polymeric doxorubicin delivery system, consisting of a PLGA core, coated with a poly-L-ornithine-FUC shell, using a layer-by-layer self-assembly method. The encapsulation efficiency and loading efficiency were 35–45% and 2–5%, respectively. The particle size was 165–180 nm and the negative ζ-potential was −24 mV. The obtained systems showed prolonged release of doxorubicin (30% in 12 h). In vivo experiments confirmed that doxorubicin-loaded multilayer particles were successfully internalized into breast tumor cells (MCF-7 cell line) and detected in the cytoplasm after 4 h incubation.

### 4.2. Chemical Modification of FUC

Chemical modification of macromolecules is another effective strategy to add desired functions and properties to them, thus overcoming some disadvantages inherent in any polymer [174]. For example, the introduction of an SH group improves the mucoadhesive properties of polymers [175]. Chen et al. [95] developed a technique to prepare self-assembled PECs from polycationic arginine-modified CS (initial CS MW 35 kDa and DAA > 85%) and thiolated FUC to enhance curcumin absorption in the intestine. For this purpose, curcumin was dissolved in aqueous ethanol and mixed with an aqueous solution of arginine-CS, and then the resulting mixture was added to the thiolated FUC solution; the encapsulation efficiency was about 60%. The formed PECs had a hydrodynamic size of about 170 nm, a PDI of 0.22, and a positive ζ-potential of +26 mV. The polymeric NPs exhibited pH-sensitive drug release; approximately 20% and 60% of curcumin was released within 25 h at pH 2.0 and 7.4, respectively. Permeability studies in a Caco-2 cell model showed that the developed polymeric systems increased paracellular permeability.

To overcome the instability of FUC-based PECs at pH ≤ 2, FUC can be modified with different strong acidic substituents. For example, Wu et al. [91] developed an oral delivery system for berberine based on FUC-taurine conjugate (synthesized from FUC with MW 80 kDa) and CS (MW 60 kDa, DDA 85%). Modification of FUC with taurine increased the negative charge density on FUC (since the sulfonic acid group of taurine is a strong acid with pKa 1.5); in addition, taurine inhibited the lipopolysaccharide-induced release of various inflammatory factors. To obtain NPs, the reverse mixing process (a mixture of berberine-CS was added to a solution of FUC) was used, colloidal stable NPs were formed at a 1.3–4-fold mass excess of taurine-FUC, and aggregation was observed at a polymer ratio of 1:1. As the mass fraction of CS in the system was increased, the average size of the NPs increased significantly from 150 to 350 nm, and the encapsulation efficiency increased from 32 to 63%, while the ζ-potential varied from −13 to +16 mV. The release of berberine from the NPs was gradual (cumulative release was 80% for 12 h) in simulated intestinal fluid (SIF, pH 7.4), while the NPs were more stable in simulated gastric fluid (SGF, pH 2.0) (20% for 12 h). The developed nanocarriers effectively inhibited the production of NO and the expression of TNF-α protein by LPS-activated macrophages.

Hydrophobic modification of polymers is an attractive method to synthesize amphiphilic macromolecules that can self-assemble in aqueous solution to form colloidal stable nanostructures with a hydrophobic core and a hydrophilic shell; various hydrophobic drugs can be loaded into the hydrophobic part of such nanosystems [176,177,178]. As a result, the pharmacodynamics and pharmacokinetics of drugs in vivo, as well as their bioavailability and toxicity profile, are improved, targeting efficiency and penetration into target cells are enhanced, and therapeutic efficacy is maximized [179]. The gonane structures (e.g., cholesterol, dexamethasone, deoxycholic acid, etc.) and a number of fatty acids (including oleic acid) are widely used as hydrophobic moieties [180,181,182,183,184]. There are not many studies on the grafting of hydrophobic moieties onto FUC. For example, Phan et al. [185] synthesized an amphiphilic conjugate of FUC-oleic acid to load poorly water-soluble drugs. The resulting derivatives were self-assembled in aqueous media into polymeric particles capable of loading hydrophobic antitumor drugs (e.g., curcumin and paclitaxel). The resulting drug delivery systems exhibited prolonged and pH-sensitive drug release. For example, curcumin was released more rapidly at pH 4.5 than under physiological conditions (pH 7.4), facilitating delivery and maximizing its effect on cancer cells.

## 5. Characterization Methods of Fucoidans and Fucoidan-Based Drug Delivery Systems

### 5.1. Characterization of Fucoidans

The members of the FUC family, despite their prominent biological activity, have not yet become regulated therapeutic agents. The structural heterogeneity, randomness of their sulfation, and branching prevented the establishment of clear relationships between FUC bioactivity and structure. The majority of FUC-based nanocarriers were based on commercial samples, without detailed analysis of the composition and architecture of native FUC. Thus, the correlations between the properties of native FUCs and the properties of their drug delivery systems remain unclear.

First of all, we remind the readers of the works on the characterization of native FUC. A detailed characterization of FUC by FT-IR and Raman spectroscopy was reported in [186,187]. The results were compared with those obtained by high-performance liquid chromatography with refractive index detector (HPLC-RI) and chemical methods (fucose method and dye method). Peak assignments of different 2D NMR sequences (COSY, HSQC, and HMBC) are given in [187]. 

The monosaccharide analysis of polysaccharides starts with the depolymerization of glycosidic bonds; the monomers are analyzed by high-performance liquid chromatography, high-performance anion-exchange chromatography, gas chromatography, capillary electrophoresis, thin-layer chromatography, and NMR [188].

Careful procedures for the extraction and purification of FUC, as well as several methods for chemical analysis of FUC composition, have been reported in [49], where FUC has been studied by mass spectrometry, SEC-MALS, capillary viscometry, Raman and NMR spectroscopy.

In addition to the useful recent review on the characterization of FUC [51], we should emphasize the importance of characterizing the branching of FUC, because the architecture of the polyion plays a significant role in PEC formation, as has been shown by molecular modeling of complexation between linear and branched polyelectrolytes, such as stars and brushes [189]. Nevertheless, the architecture of FUCs has rarely been analyzed in the study of their complexation with polycations.

Although the degree of branching of different FUCs has been determined by 2D NMR [49,50,190,191,192], knowledge of the total number of nodes is not sufficient to discriminate between comb-like and 3D branching. On the other hand, the hydrodynamic properties of macromolecules strongly depend on their architecture. For example, branched macromolecules have a lower intrinsic viscosity [η] than linear macromolecules of the same molecular mass. This is a problem when using SEC to determine the molecular mass of branched polymers, because linear macromolecules are used as standards in SEC. The dimensionless ratio of hydrodynamic radii of equivalent spheres, calculated from capillary viscometry *R*_visc_ = (3[η]*M*/10π*N*_A_)^1/3^ and DLS (*R*_h_ = *kT*/6πη*D*), can be a robust and useful parameter, because it depends on macromolecular rigidity and branching. In these formulas, *M* is the molecular mass, *N*_A_ is the Avogadro’s number, *k* is the Boltzmann constant, *T* is the temperature, η is the solvent viscosity, and *D* is the translational diffusion coefficient. Originally, the dimensionless ratio *R*_visc_/*R*_h_ was expressed in terms of the so-called hydrodynamic invariant *A*_o_ = *D*η(*M*[η])^1/3^/*T* [193]. If *A*_o_ is expressed in erg grad^−1^ mol^−1/3^, then *R*_visc_/*R*_h_ = 3.43 × 10^9^ *A*_o_. The hydrodynamic invariant has been experimentally determined for a number of homologue series of synthetic polymers of different architectures. According to these measurements, we can deduce that the average *R*_visc_/*R*_h_ ratio of linear macromolecules increases with their rigidity from 1.1 to 1.3. For solid spheres, *R*_visc_ = *R*_h_. Branched macromolecules exhibit *R*_visc_/*R*_h_ < 1. In particular, *R*_visc_/*R*_h_ < 1 was observed for FUC extracted from *F. vesiculosus* [194]. Another structure-sensitive ratio is determined by light-scattering techniques—it is the ratio of macromolecular radii obtained by static and dynamic light-scattering, *R*_g_/*R*_h_. The *R*_g_/*R*_h_ ratio depends on the shape of the scattering object and the density distribution within it. For example, *R*_g_/*R*_h_ = 0.78 corresponds to spheres of constant density; *R*_g_/*R*_h_ < 0.78—to nanogels or core–shell micelles with higher density in the center, *R*_g_/*R*_h_ of moderately dispersed linear polymers in good solvents is about 2 [195]. The magnitude of *R*_g_/*R*_h_ of FUCs extracted from *L. japonica* [50] and *F. vesiculosus* [194] corresponded to their spherical shape. FUC extracted from *F. vesiculosus* was considered to be a hyperbranched polymer, because its angular dependence of light-scattering intensity corresponded to the theoretical function developed for the ABC-polycondensation model. The fitting of the branching parameter provided the number of branching points per FUC macromolecule (molecular mass between knots for differently hydrolyzed FUCs extracted from *F. vesiculosus* was determined in the range of 16–60 kDa) [194].

As the rheological behavior of polymers is sensitive to their architecture, it can serve as an indirect indicator of the branching of FUC. Low-weight FUC extracted from the sea cucumber *Apostichopus japonicas* (Aj-LWM) contained regularly repeating branched units, as studied by a combination of infrared spectroscopy, methylation analysis, enzymatic degradation, and nuclear magnetic resonance [196]. The shear rate dependence of the apparent viscosity of Aj-LWM was significantly different from that of linear FUC extracted from the sea cucumber *Acaudina molpadioides* [196]. The different rheological behavior of FUCs extracted from different sources implies different architectures of FUCs, as pointed out in the review [197].

When combining FUC with other polycations or polyampholytes, one should pay attention to the presence of helical conformers in FUC, because a higher level of structural organization of PEC components is favorable for the construction of various nanodevices, nanocontainers, and nanoreactors [189]. The possibility of helical conformations of FUC has been shown in [52,198,199].

### 5.2. Characterization of FUC–CS Complexes

Most of the FUC-based PECs belong to the PEC of FUC with CS. Since the hydrophobic groups of CS and FUC strongly affect the aggregation of the original polymers in aqueous solutions, the degree of acetylation of CS and the degree of methoxylation of FUC should be measured before mixing their solutions.

The -OSO_3_^−^ groups of FUC are negatively charged, regardless of pH, whereas the charge of the amino groups of CS depends on pH (they are positively charged below pH 7). The electrostatic interactions between FUC and CS were analyzed using the relative charge density model [83]. The model predicts the distribution of both positive amino groups and negative sulfate ions involved in polyelectrolyte complexation, in terms of pH variations in the range of 2 ≤ pH ≤ 6 and mass ratios of FUC/CS. 

The most commonly used methods applied in characterization of FUC–CS complexes are listed in Table 1.

Polysaccharide structure is probed in real space by microscopy and XRD, and in Fourier space, by scattering, and FT-IR spectroscopy. ELS, TEM, SEM, FT-IR, XRD, and NTA methods listed in Table 1 cover a wide range of length scales. In electron microscopy, the length scale is the wavelength of an electron. It depends on the acceleration voltage (0.017 nm for 5 kV of acceleration voltage, and 0.0027 nm for 200 kV of acceleration voltage). The copper Kα wavelength of 0.154 nm is used in X-ray diffraction. Light-scattering provides much larger length scales. Its length scale (1/*q*) varies with the scattering angle θ (the wavelength *q* = 4π*n*sin(θ/2)/λ, where *n* is the refractive index of the solvent, θ is the scattering angle, and λ is the wavelength of the incident light). For aqueous solutions at λ = 632.8 nm, the variation of θ from 40 to 140° allows to change the length scale in the range from 10 to 150 nm.

Since solutions become opaque as the polyanion:polycation charge ratio approaches unity, the most popular and robust method for controlling PEC formation is turbidimetry. Turbidity is determined as τ = −log*T*/*b*, where *b* is the path length and *T* is the transmittance, reduced by scattering. One component of the PEC is titrated with another to a turbidimetric endpoint (precipitation). At non-stoichiometric ratios, polyelectrolytes of significantly different MWs produce soluble PECs [200]. The size of the PECs, and thus their turbidity, increases as the charge equilibrium is approached. PEC precipitate at the stoichiometric endpoint, which can be identified as the point of equality of positive and negative charges in the mixture.

Using the ELS, four important parameters can be obtained: the hydrodynamic radius *R*_h_ of the particles (this is the DLS data), the polydispersity of their distribution (if it is unimodal), the ζ-potential (the ELS data), and the light-scattering intensity (*I*). DLS gives the intensity-averaged *R*_h_ corresponding to the maximum of the *I*(*R*_h_) distribution. Typically, only apparent *R*_h_ are obtained at one scattering angle and one concentration (true *R*_h_ should be obtained by double extrapolation of apparent *R*_h_ to zero scattering angle and zero concentration). Because *I* is extremely sensitive to larger scatterers, signals from smaller but more abundant species may be obscured by stronger scattering from larger particles. In the case of multimodal *R*_h_-distributions, the largest peak of the slowest mode corresponds to a tiny number of large aggregates, due to the extreme sensitivity of *I* to larger scatterers. The concentration of polysaccharides should be chosen with respect to their light-scattering intensity, which should fall within a recommended diapason. Instead of turbidimetry, the approach to charge equilibrium can be monitored by the growth of *I* or *R*_h_. 

The ζ-potential is the measure of the effective electric charge on the NP surface. The higher the value of ζ-potential, the more charges that are present on the particle surface, indicating stronger electrostatic repulsion between particles. The absolute value of the ζ-potential, above which colloidal stability is ensured, is 30 mV (the ζ-potential can be positive or negative, depending on the surface charge). The sign of the PEC ζ-potential indicates which component is on the surface. 

NTA measures the size of NPs by computer analysis of the trajectories of their Brownian motion. The presence of small amounts of large particles does not affect the accuracy of NTA measurements. Because NTA provides a number distribution, it allows relative particle concentrations to be determined, unlike DLS. NTA can follow the Brownian motion of any component in the mixture, provided that component is fluorescently labeled. The maximum concentration that can be measured by NTA is 10^9^ particles per mL. Thus, NTA can detect solutions that are 10–1000 times more dilute than those measured by DLS. R_h_ obtained by DLS from intensity averaged R_h_ distributions should be higher than number averaged Rh obtained by NTA. The limitations of NTA were pointed out in [201]. NTA was used by Oliviera et al. [56] to measure the size of FUC-CS particles by directly monitoring the Brownian motion of laser-illuminated particles in suspension (116 nm). This size was slightly smaller than the hydrodynamic diameter measured by DLS (141 nm).

FT-IR is a very popular and informative method for PEC characterization. Since IR absorption depends on the interaction of infrared radiation with vibrating dipole moments of molecules, it is particularly sensitive to the groups involved in polyelectrolyte complexation. The intensity of infrared peaks depends on the change in dipole moment, with respect to bond length during a molecular vibration. The higher the polarity of the bond, the higher the IR absorption. In particular, the adsorption of IR radiation by the carbonyl bond is high because this bond is highly polar, due to the large electronegativity difference between carbon and oxygen. The position of the C=O bond of the primary amide of CS (1650 cm^−1^) became a marker of its interaction with FUC. In fact, this band was red-shifted from the initial value of 1650 cm^−1^, due to intermolecular interactions (hydrogen bonding or electrostatic interactions) within FUC/CS complexes. The positions of the other characteristic peaks of CS (-NH_3_^+^ at 1560 cm^−1^, C-O-C at 1150 cm^−1^, and C-O at 1026 cm^−1^) and FUC (S=O at 1160–1260 cm^−1^ and C-O-S at 845 cm^−1^) remained unchanged during complexation [80,93,99]. The bands of C-O-C, S=O, and C-O-S groups of fucoidan shifted from 1426 cm^−1^, 1028 cm^−1^ and, 883 cm^−1^, to 1406 cm^−1^,1022 cm^−1^, and 878 cm^−1^, respectively, in NPs formed by low molecular weight FUC with CS [128].

TEM is the most popular method used to characterize the morphology of PECs. Since TEM magnification depends on the acceleration voltage, it is important to use low-dose techniques that minimize the structural reorganization of organic materials that inevitably occurs during electron beam irradiation. A 10-fold increase in magnification results in a 100-fold increase in the delivered electron dose [202], which can damage the sample. A review of TEM methods and sample preparation for TEM can be found in [203]. 

The application of XRD is suitable for studying PEC, whose initial components are crystalline; then, XRD can follow the changes in crystallites produced by complexation. Such a study was successfully carried out in [126] for curcumin-loaded PEC of FUC-CS. Among the three components of FUC-CS curcumin particles, two initial components, curcumin and CS, were crystalline. The XRD reflections of curcumin crystals embedded in the PEC of FUC-CS were weaker than those obtained by directly mixing FUC, CS, and curcumin powders in the same ratio, indicating the interaction between curcumin and the PEC matrix. In this work, the hydrolyzed CS was used. The hydrolyzed CS showed its own crystalline peaks which disappeared in the prepared PEC, suggesting that the successful formation of PEC also destroys the crystalline structure of the original CS.

### 5.3. Characterization of FUC-Based Drug Delivery Systems Containing Proteins and Peptides

The FUC-CS complexes suffer from a limited pH range, because CS becomes insoluble at pH > 7. To extend the pH range of FUC-based NPs, FUC has been combined with globular proteins and polypeptides. Since native proteins and polypeptides have different types of organized structures (such as α-helices or β-sheets), the range of methods for characterizing their drug delivery systems is sufficiently extended, as shown in Table 2.

Table 2 shows that FUC was mainly blended with zein obtained from corn. The hydrophobicity of zein prohibits its solubility in water, but zein is still soluble in water-ethanol solutions. Thus, the core-shell particles with hydrophobic zein cores surrounded by the polysaccharide shell can be obtained by the anti-solvent precipitation method (dropwise addition of ethanol solutions of zein into aqueous solutions of polysaccharides) [206]. The zein-based NPs coated with a wide range of polysaccharides (including CS, alginate, pectin, gum arabic, etc.) were described in [197], but the case of zein-FUC NPs was not considered. 

The last two rows of Table 2 concern the interaction of FUC with proteins. The complexation of β-lactoglobulin (BLG) with FUC was studied in the range of pH 3.0–6.5 [204]. The conformational stability of BLG in the complexes decreased significantly at pH < pI 5.2 (when the components were oppositely charged), but increased slightly at pI ≤ pH (when both components of the complex were negatively charged, but BLG formed dimers with a large dipole moment).

Interpolymeric complexes of bovine serum albumin (BSA) and FUC were studied in [205] at a weight ratio of 5:1 in the pH range between 2.5 and 8.0. BSA is a model globular protein with a molecular weight of approximately 66.5 kDa. When BSA is positively charged at pH < pI 4.9, the positive charges of BSA and the negative charges of FUC (–NH_3_^+^ and −OSO_3_^−^) favor the formation of BSA-FUC complexes with very dense and compact internal structures. The turbidity of the complexes increased drastically at pH 4.5, and the maximum biopolymer interactions were induced at pH 4.0. Increasing NaCl concentration (from 0 to 1 M) shifted the critical pH to a more acidic value, which can be explained by salt screening. At 0.01 M NaCl, the aggregated BSA-FUC complexes dissociated into a soluble state at pH 4.5.

Complexation of FUC with peptides resulted in associative phase separation (coacervation), which developed with increasing peptide: FUC weight ratio [82]. The coacervation mechanism was proposed based on the ITC results. Before discussing the ITC results, a few words about coacervation: Coacervates are formed during the associative phase separation of oppositely charged molecular species, such as polyions, colloids, and polyampholytes.

Complex coacervation is formed at relatively low concentrations (<3 to 4 wt% total solids) and ionic strengths below 0.4 M [207]. During coacervation, the solutions visibly separate into two regions. The opaque phase of coacervates forms at the bottom, and the transparent supernatant is the equilibrium liquid that is predominantly free of these species. Coacervates exist as spherical droplets, often on the nanometer or micron scale in diameter, resembling an emulsion, except that they do not necessarily contain stabilizing molecules, such as surfactants. With advances in drug delivery, the use of coacervates as controlled delivery vehicles has only recently begun. Self-assembly in water and affinity to proteins, which significantly reduce protein denaturation and burst release, make coacervates a rational choice for controlled release of proteins and peptides [208].

#### 5.3.1. Fluorescent Spectroscopy 

The intrinsic fluorescence of protein residues, such as tryptophan, is used as a reporter of the chemical environment of the protein. Either the magnitude of the emission intensity, or the shift in the wavelength of the maximum emission intensity, can probe the complexation of the protein. 

The fluorescence spectroscopy of zein is based on the fluorescence of its tyrosine residues. When excited with 280 nm light, zein exhibits emission at 309 nm. In the FUC-based NPs, the fluorescence intensity of zein was significantly decreased, suggesting that the interaction of zein with the components of the NPs could lead to intrinsic fluorescence quenching of zein [10,164]. The quenching of zein’s fluorescence was observed in core-shell NPs formed by antisolvent precipitation of alcohol solutions of zein onto the aqueous solutions of other polyanionic polysaccharides, such as carrageenan [209] and propylene glycol alginate [210].

#### 5.3.2. Fourier-Transform Infrared Spectroscopy

The most important characteristic FT-IR bands of proteins are the amide I and II bands, as they are sensitive to the secondary structure of the protein [206]. The amide I peak position (mainly C=O stretching) occurs in the region of 1700 to 1600 cm^−1^, and the amide II band occurs in the region of 1600 to 1500 cm^−1^ (C-N stretching, coupled with N-H bending mode). The changes in the amide I and amide II bands generally indicate the transformation of the secondary structure of zein, as well as the appearance of hydrogen bonds. The shift of the OH stretching band (3100–3500 cm^−1^) to higher frequencies is usually due to hydrogen bonding. The bands at 3000 and 2800 cm^−1^ can give information about hydrophobic forces. 

Examples of shifts of the main bands in FUC-zein NPs entrapping curcumin (Cur) observed in [36] are listed in Table 3. The interaction between zein and FUC was revealed by the blue shift of the amide I peak in zein-FUC NPs entrapping curcumin [36], and also in zein–FUC NPs entrapping quercetin [164].

#### 5.3.3. Crystallinity

When the delivered small molecule drugs are crystalline, the character of their entrapment within the PEC particles can be followed by XRD or DSC. 

For example, in the resveratrol-loaded PEC of zein-FUC, both pristine polyelectrolyte components (zein and FUC) were amorphous, but the pristine low-molecular-weight drug resveratrol was crystalline [152]. The disappearance of the sharp XRD diffraction pattern of resveratrol indicated its molecular entrapment in the zein-FUC complexes. 

Another low-molecular-weight crystalline drug (curcumin) also lost its crystallinity within the zein-FUC complexes, as indicated by the disappearance of its exothermic DSC peak [36].

#### 5.3.4. Circular Dichroism 

Since folded globular proteins have unique CD signatures in their native state, CD can be a convenient method to evaluate the retention, loss, or modification of protein structures. The influence of complexation of proteins (and/or polypeptides) with FUC on their secondary structure has been revealed using CD spectroscopy. 

The CD study of conformational changes of proteins and peptides upon their interaction with FUC is facilitated by the absence of distinct CD peaks of FUC. The secondary structure of individual zein particles has been studied by CD spectra, showing two negative peaks at 209 nm and 223 nm, and a positive peak at 193 nm [163,211,212,213] containing α-helix, β-sheet, and β-turn. The changes of the corresponding CD maxima of zein in its PECs with FUC [153], hyaluronic acid [211], and propylene glycol alginate [203] suggested the changes in the secondary structure of zein were induced by its interactions with polysaccharides. In particular, the α-helix content of zein decreased after binding with the polysaccharides, while the content of the β-sheet and β-turn increased. 

CD revealed that the conformation of protamine changed from a random coil to an α-helix after the formation of the protamine—FUC complex [82]. The same conformational transition from random coil to α-helix was observed by CD for the complexation of the cell-penetrating peptide TPP1880 with FUC [199].

#### 5.3.5. Isothermal Titration Calorimetry

In the ITC, the composition of the system is changed by injecting a series of small aliquots of the ligand solution into the sample cell. The raw thermogram contains the enthalpy changes (Δ*H*) at different stoichiometries of the components. Typically, a titration curve with sigmoidal tendency is able to obtain the precise fit to an appropriate binding model, which gives the binding constant (*K*_b_); the number of proteins bound per PE chain (*N*); the reaction entropy, Δ*S*_r_, and Gibbs energy, Δ*G*_r_ [214].

In the case of multiple binding events with their own H, the binding isotherm will be a sum of these events. The total enthalpy of mixing, Δ*H*, provides insight into the role of hydrophobic, H-bonding, electrostatic interactions, and conformational changes of macromolecules, while the entropy of binding, Δ*S*, reflects the changes in restriction/freedom of the backbone and side chain atoms and the rearrangement or release of solvent water molecules and ions. Protein folding and drug action [215], as well as coacervation [216], are responsible for changing the binding enthalpy. The problems of ITC are discussed in [217,218].

The electrophoretic studies of complex formation can be helpful in providing data on the charge and colloidal stability of PEC particles, which perfectly complement the ITC data. It is important to check whether a given protein is modified upon interaction with the polyelectrolyte. In this case, part of the calorimetric signal would be due to partial denaturation or refolding of the protein.

The ITC curve for the complexation between FUC and protamine showed several steps of complexation [82]. The first step was ion pairing; the second step was complex coacervation. In the first step (at protamine-FUC weight ratio less than 0.8), the *K*_b_ was high (2.07 × 10^6^), indicating the formation of a strong interaction between protamine and FUC. The observed exothermic enthalpy (−28.1 kJ/mol) could result from the protamine-FUC association. FUC has multiple binding sites for protamine (*N =* 7.66), suggesting non-specific binding between protamine and FUC molecules. In the second reaction step (at weight ratio greater than 0.8), the binding affinity decreased (5.25 × 10^4^), indicating that the interaction became much weaker. The higher binding number (*N* = 10.0) and exothermic enthalpy (−111.2 kJ/mol) were mainly caused by agglomeration of the protamine/FUC nanocomplex formed in the first stage.

The ITC curve for the complexation between low molecular weight FUC and cell-penetrating peptide TPP [199] also showed that the heat evolved and adsorbed during two stages: (i) formation of TPP:LMWF ion pairs, and (ii) complex coacervation. The binding stoichiometry number (*N*) was 1.73 in the first reaction step. This value indicates that TPP binds to LMWF in a non-specific binding mode (multiple binding sites). In the second reaction step (complex coacervation), *N* increased to 13.2, due to shielding of base groups of TPP within the coacervate.

## 6. Conclusions and Future Perspectives

The marine polysaccharide FUC has a great structural and chemical diversity, as well as its own biological activity. Due to the presence of the -OSO_3_H group with strong acidic properties, FUC macromolecules are able to form PECs with different polycations, which are of interest as polymeric drug delivery systems with modified (prolonged, controlled, and targeted) release. The formed PECs are characterized by a variety of physicochemical and biopharmaceutical properties; however, the following key strategies can be highlighted for their biomedical applications:

(i) development of oral drug delivery systems based on PECs of FUC with CS that exhibit pH-dependent modified drug release, due to deprotonation of CS amino groups at pH above 6.5;
(ii) development of targeted drug delivery systems (mainly anti-tumor agents and nanoantibiotics) based on the specific affinity of the FUC molecule to the cell adhesion molecule P-selectin, which is overexpressed in tumor tissues, as well as to other biological target structures (including macrophages);
(iii) design of polymer systems to improve the bioavailability of poorly water-soluble drugs by loading hydrophobic substances into FUC-based PECs with CS, dendrimers, zein, as well as into multilayer structured “hydrophobic core-hydrophilic shells” and into self-assembling NPs, based on hydrophobically modified FUC;
(iv) chemical modification of FUC to introduce substituents with desired functions, e.g., thiol moieties enhance mucoadhesive properties, taurine moieties enhance acidity, and grafted hydrophobic fatty acids cause the formation of self-assembling micelle-like structures in an aqueous medium suitable for hydrophobic drug delivery.

Pharmaceutical development of innovative polymer delivery systems based on FUC is a complex technological process that requires careful selection of conditions for PEC formation (including polymer concentrations and their mass ratios, pH of polymer solutions, mixing order of components). In addition, careful characterization of FUC is important, since the structure of FUC, its MW and degree of sulfation, as well as the monomeric composition and branching, will have a significant impact on the process of PEC formation and its properties. Unfortunately, not all researchers report the full characteristics of the FUC used, which makes the comparison and further application of the reported results difficult.

The study of the properties of formed PECs (primarily their shape, hydrodynamic size, ζ-potential, and colloidal stability) is also of key importance for pharmaceutical development, so it is necessary to use a set of methods for their comprehensive evaluation. More extensive use of accurate and reliable methods, such as static light-scattering, nanoparticle tracking analysis, and isothermal titration calorimetry, is recommended, in addition to the traditional methods of NP size and shape assessment, dynamic light-scattering, and scanning electron microscopy.

## Figures and Tables

**Table 1:** Methods applied for the characterization of FUC–CS complexes.

Polysaccharides		Delivered Compounds	Methods of Characterization	References
Fucoidan	Chitosan			
*F. vesiculosus* 50–190 kDa	190–310 kDa	Quercetin	FT-IR, ELS, TEM	Barbosa et al. [99]
*F. vesiculosus* 50–190 kDa	50–190 Da	Methotrexate	FT-IR, DLS, TEM	Coutinho et al. [80]
*F. vesiculosus* 80 kDa	DDA > 75% 35 kDa	99mTc-methylene diphosphonate	FTIR, ELS, TEM	Huang et al. [93]
*F. evanescens* (123 and 340 kDa) *S. cichorioides* (773 kDa)	DDA 94%	-	NMR 1D (1H, 13C) and 2D (COSY, ROESY, HSQC, HMBC) ITC, ELS, Molecular modelling	Rasin et al. [52]
*L. japonica*	Hydrolyzed CS 61 kDa, DDA 91.4%	Curcumin (ethanol)	FT-IR, XRD, ELS, TEM	Don et al. [126]
*F. vesiculosus* 45–75 kDa	40–150 kDa	Gemcitabine	ELS, NTA, SEM, TEM	Oliviera et al. [56]
*F. vesiculosus*	DDA 75–85%		potentiometry	Lee et al. [83]
*L. japonica* 500–1500 Da	50–190 Da DDA 75–85%	Conjugated dyes	FTIR, SEM, ELS	Wu et al. [128]
*F. vesiculosus*	DDA > 75%		FTIR, TEM, ELS	Liu et al. [127]
*F. vesiculosus* 58.3 kDa	quaternary chitosan 35 kDa DDA ≅ 85%	Epigallocatechin gallate	FT-IR, TEM, ELS	Huang et al. [135]
*F. vesiculosus*	N-(2-hydroxy-3-trimethylammonium) propylchitosan		ELS, turbidimetry	Chuang et al. [137]

ELS—electrophoretic light-scattering; TEM—transmission electron microscopy; FT-IR—Fourier-transform infrared spectroscopy; XRD—X-ray diffraction; NTA—nanoparticle tracking analysis; ITC—isothermal titration calorimetry.

**Table 2:** Methods of characterization of NPs, obtained by mixing FUC with proteins and/or polypeptides.

Components of PEC		Delivered Compounds	Methods of Characterization	Reference
Fucoidan	Protein, Polypeptide			
*L. japonica* 50–200 kDa	zein	Quercetin	ELS, FS280 FT-IR, TGA, XRD, SEM	Zhang et al. [164]
*L. japonica* 50–200 kDa	zein	Curcumin	EDLS, FT-IR, SEM, TEM, DSC	Zhang et al. [36]
*L. japonica* 120 kDa	zein	Resveratrol	EDLS, SEM, FS280, CD, FT-IR, XRD	Liu et al. [10]
*L. japonica* 120 kDa (+Sodium caseinate)	zein	Pterostilbene	EDLS, TEM, FT-IR, XRD	Liu et al. [151]
*L. japonica* 80 kDa	Protamine (5 kDa) Cy3/Cy5-labeled protamine		Turbidimetry, ITC DLS, TEM, FT-IR, CD, FS350	Lu et al. [82]
Hydrolyzed FUC (8775 Da)	Cell-penetrating peptide TPP (1880 Da) Random coil Cy5-labeled TPP		Turbidimetry, FT-IR, CD, ITC, DLS, ELS	Cheng et al. [199]
*F. vesiculosus*	β-Lactoglobulin (pI 5.2)	-	Nephelometry, DSC, sedimentation velocity	Burova et al. [204]
*Undaria pinnatifida* *656 KDa*	Bovine serum albumin		Turbidimetry, EDLS, Viscometry	Kim et al. [205]

FSλ—fluorescent spectroscopy (λ is the excitation wavelength in nm); CD—circular dichroism; DSC—differential scanning calorimetry.

**Table 3:** FT-IR bands (cm^−1^) observed in zein–FUC and zein–FUC-Cur particles [36].

Vibration Type	Components of PEC	zein–FUC	zein–FUC-Cur
OH-stretching (FUC and zein)	3427 (zein), 3447 (FUC)	3448	3443
S=O stretching (FUC)	1256	1256 ↓	1256 ↓
C-O-S stretching (FUC)	844	843	841
Amide I (zein)	1646	1651	1648
Amide II (zein)	1546	1544	1547

↓ indicates reduced intensity.

## Data Availability

Not applicable.
